# Modeling R_0_ for Pathogens with Environmental Transmission: Animal Movements, Pathogen Populations, and Local Infectious Zones

**DOI:** 10.3390/ijerph16060954

**Published:** 2019-03-17

**Authors:** Jason K. Blackburn, Holly H. Ganz, José Miguel Ponciano, Wendy C. Turner, Sadie J. Ryan, Pauline Kamath, Carrie Cizauskas, Kyrre Kausrud, Robert D. Holt, Nils Chr. Stenseth, Wayne M. Getz

**Affiliations:** 1 Spatial Epidemiology and Ecology Research Laboratory, Department of Geography, University of Florida, 3141 Turlington Hall, Gainesville, FL 32611, USA; 2 Emerging Pathogens Institute, University of Florida, 2055 Mowry Road, Gainesville, FL 32611, USA; sjryan@ufl.edu; 3 Davis Genome Center, University of California, 451 Health Sciences Dr., Davis, CA 95616, USA; holly.ganz@mac.com; 4 Department of Biology, University of Florida, Gainesville, FL 32611, USA; josemi@ufl.edu (J.M.P.); rdholt@ufl.edu (R.D.H.); 5 Department of Biological Sciences, State University of New York, 1400 Washington Avenue, Albany, NY 12222, USA; wcturner@albany.edu; 6 Quantitative Disease Ecology & Conservation Lab, Department of Geography, University of Florida, 3141 Turlington Hall, Gainesville, FL 32611, USA; 7 School of Life Sciences, University of KwaZulu-Natal, Durban 4041, South Africa; 8 School of Food and Agriculture, University of Maine, 5763 Rogers Hall, Room 210, Orono, ME 04469, USA; pauline.kamath@maine.edu (P.K.); wgetz@berkeley.edu (W.M.G.); 9 Department of Environmental Science, Policy, and Management, University of California, Berkeley, 130 Mulford Hall, Berkeley, CA 94720, USA; cizauskas@gmail.com; 10 Centre for Ecological and Evolutionary Synthesis (CEES), Department of Biosciences, University of Oslo, P.O. Box 1066 Blindern, 0361 Oslo, Norway; kyrre.kausrud@gmail.com (K.K.); n.c.stenseth@ibv.uio.no (N.C.S.); 11 School of Mathematical Sciences, University of KwaZulu-Natal, Durban 4041, South Africa

**Keywords:** basic reproductive number (R_0_), indirect disease transmission, disease emergence, disease control, pathogen spillover, animal movement

## Abstract

How a disease is transmitted affects our ability to determine R_0_, the average number of new cases caused by an infectious host at the onset of an epidemic. R_0_ becomes progressively more difficult to compute as transmission varies from directly transmitted diseases to diseases that are vector-borne to environmentally transmitted diseases. Pathogens responsible for diseases with environmental transmission are typically maintained in environmental reservoirs that exhibit a complex spatial distribution of local infectious zones (LIZs). Understanding host encounters with LIZs and pathogen persistence within LIZs is required for an accurate R_0_ and modeling these contacts requires an integrated geospatial and dynamical systems approach. Here we review how interactions between host and pathogen populations and environmental reservoirs are driven by landscape-level variables, and synthesize the quantitative framework needed to formulate outbreak response and disease control.

## 1. Introduction

Environmental factors play a decisive role in the emergence of infectious diseases, particularly when indirect transmission is involved. Briefly, pathogen transmission can be defined as direct when an infected individual can infect another, such as classical influenza air-borne transmission or HIV sexual transmission. Indirect transmission occurs when the pathogen is acquired from the environment, such as grazing on pathogen-contaminated feed, or a vector, such as an insect, infecting one individual after feeding on another infected individual. Estimating indirect transmission requires that we evaluate the impact of environmental factors on the basic reproductive number, R_0_, of a pathogen, as has been done in the context of the recent Ebola outbreak in Africa [1], and mosquito-transmitted Zika virus [2]. The number of new cases from a single case, the R_0_, has long been the basis for assessing outbreak severity and control impacts. Here we provide a brief overview of R_0_ before providing an overview of challenges to these calculations for pathogens that persist in the environment for long periods. 

### 1.1. A Brief History of R_0_

1925–1975: R_0_ is central to our understanding of both population growth and the spread of disease. As reviewed by Heesterbeek [3], Dublin and Lotka [4] were the first to cast R_0_ as “… *the number of female offspring born to one female during her lifetime*.” In the context of epidemiology, Dietz [5] defined R_0_ as “*… the number of secondary cases that one case can produce if introduced in a susceptible population.*”

1976–1990: The role of R_0_ in epidemiology received a major boost in 1982 at a conference on *Population Biology of Infectious Disease* in Dahlem, Germany (R. M. Anderson and R. M. May, organizers). Calculating R_0_ from data became a major challenge in the 1980s due to inhomogeneities, such as variation in individual susceptibility. This problem was solved by Diekmann et al. [6] in providing a method of calculating a *next generation matrix* whose dominant eigenvalue is R_0_. Using R_0_, however, to determine the actual growth rate of an epidemic requires that the *generation time G* also be known. 

Modern demography: If *l_x_* is the proportion of individuals surviving to exact age *x* and *b_x_* is the force of natality (i.e., $\int_{i}^{i+1}b_{x}dx$ is the number of female young born to each female in the interval [*i*,*i* + 1)), then:

(1) $$R_{0}=\int_{0}^{\infty }l_{x}b_{x}dx\;\mathrm{and}\;G=\int_{0}^{\infty }xl_{x}b_{x}dx/R_{0}.$$

Indirectly transmitted diseases: The next generation matrix approach of Diekmann et al. [6], applicable to directly transmitted diseases, was extended by van Driessche and Watmough [7] to class-structured population processes that accounted for both new within-class infections and the transfer of infection among classes. The structure of this approach could then be applied, for example, to calculating R_0_ in vector-host-pathogen transmission systems. 

Critique: Methods for calculating R_0_ have been criticized for conflating processes affecting disease transmission. For example, Li et al. [8] pointed to the fact that vertical transmission events may be cancelled out by disease induced mortality events in differential equation models, but not in next-generation matrix methods, leading to ambiguity in the calculation of R_0_. However, this appears to be a shortcoming of oversimplifying differential equation models of transmission, rather than next-generation matrix methods per se. This conundrum is only resolvable through more careful formulation of the way R_0_ is computed for ecologically complicated epidemics, such as those that have a determinative environmental component. Long-lived environmental pathogens present exemplar cases for challenging the R_0_ formulations currently available and require new strategies to estimate transmission. 

### 1.2. Challenges for R_0_ for Environmentally Maintained Pathogens

**Figure 1:**
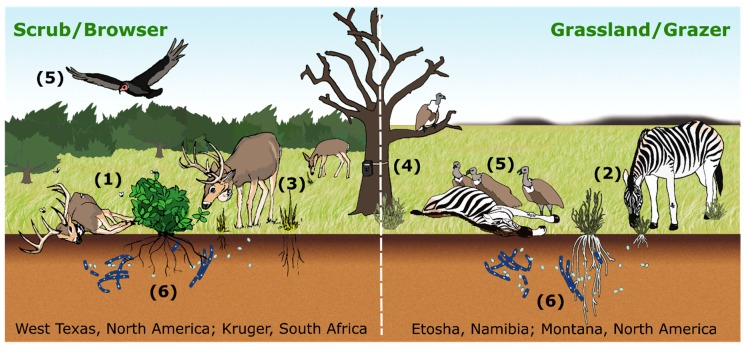
Conceptual diagram of anthrax transmission from hosts interacting with local infectious zones (LIZs) on two different landscapes. Transmission to browsers, here white-tailed deer in the scrub habitat of west Texas (left panel), can occur through ingestion of contaminated vegetation, which can be amplified by blow flies and biting flies (**1**). Grazers, here zebras in Etosha National Park, Namibia (right panel), are exposed through ingesting contaminated grasses and soils (**2**). On both landscapes, host movements are recorded with GPS collars (**3**), foraging at LIZs is captured with camera traps (**4**) and mortality is found by following vultures to carcasses (**5**). *B. anthracis* persists in soil and may have a soil-borne life cycle in both systems (**6**). Flies do not play a major role in open grassland grazing systems, particularly when vertebrate scavengers are abundant, but may in the browser systems.

For pathogens, such as *Bacillus anthracis* [9], the causative bacterium of anthrax, and chronic wasting disease (CWD) [10], that persist in spatially limited environmental reservoirs, estimates of R_0_ to date have not explicitly included their spatial structure, which can be characterized as a distribution of host carcass-generated local infectious zones (LIZs) distributed over the landscape. In such cases, exposure depends on susceptible individuals arriving at the reservoir and contacting the pathogen [11], for example through ingestion [12] or inhalation [13]. These LIZs may remain infectious for some period of time, ranging from hours (Ebola virus [14]) to weeks (*Mycoplasma bovis* [15]) to months (*Brucella abortus* [16]) to decades (*Bacillus anthracis* [17]), with subsequent exposures arising from additional naïve hosts seeking resources within the reservoir. In the case of pathogens that persist across host generations, a traditional susceptible, exposed, infected, recovered (SEIR) model [18] cannot adequately capture exposure at LIZs. *Our central tenet is that successfully modeling LIZ exposure dynamics requires an integrated geospatial and mathematical approach, tracking seasonal changes on the landscape and the effects of those changes on host movements (resource selection, site fidelity, and foraging) and pathogen persistence within LIZs* (Figure 1). 

In support of this tenet, we present a spatially explicit pathogen-reservoir-host formulation that gives rise to a natural mathematical outbreak description in which both temporal and spatial processes occurring at local and larger scales are linked in meaningful ways and is broadly applicable across several pathogen/host systems (Table 1). We show how such a formulation can help to test the idea that seasonal outbreaks contribute to the accumulation of LIZs to fuel future outbreaks. The spatial and temporal processes involved are driven by vegetation phenology ([19]; or aquatic analogs [20]), climatic variables, and host/LIZ interactions (Figure 1). 

**Figure 2:**
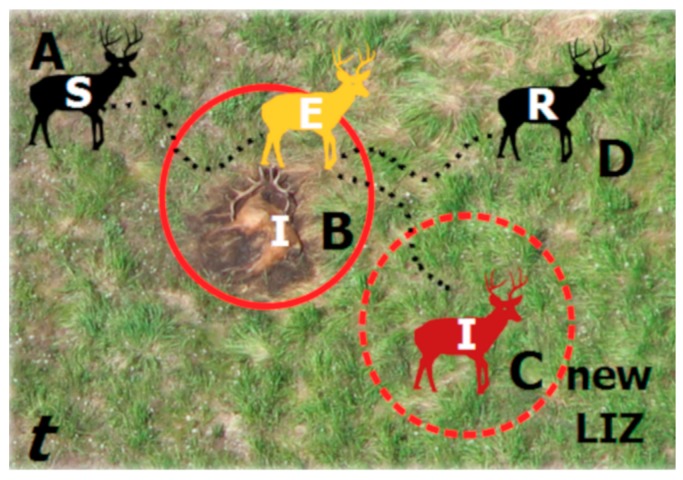
Components of an SEIR model as applied to environmental transmission during a single outbreak season (*t*). Susceptible hosts (**S**) move across the landscape (**A**) and contact infectious LIZs (**I**) and become exposed (**E**; **B**). As they leave the LIZ, they may succumb to infection and die, becoming a new LIZ, establishing in time *t* and persisting across future time periods (*t* + 1,…,*n*) (**C**) or recover (**R**) and survive to a future time period (**D**).

By way of example, in Figure 1 we illustrate anthrax transmission (as an example of environmental transmission) on two landscapes: (1) the scrub habitat of west Texas (left panel), where blow flies [33] and biting flies [34] can increase local case intensity of white-tailed deer (*Odocoileus virginianus*) predominantly browsing during the outbreak season; and (2) the grasslands of Etosha National Park in Namibia (Etosha; right panel), where grazing near LIZs during the wet season is the primary mode of transmission [11,12]. On each of these landscapes LIZ persistence has been confirmed. Furthermore, movement data and foraging behavior observations (e.g., camera traps [11]) strongly suggest the formulation of a new R_0_; in these landscapes, it is the host interaction with carcasses (both landscapes) or fly-contaminated browse (Texas scrub; left panel) that drives the exposure and subsequent infection. In Figure 2, we illustrate how such host/LIZ interactions can be compartmentalized in an SEIR framework. The formulation itself suggests that while controlling individual outbreaks may be strategically desirable, reducing the number of local infectious zones may be a better long-term strategy for disease management.

Under-studied aspects of the role of environment in pathogen transmission are the mechanisms that permit pathogens to be maintained in reservoirs [35,36]. Some pathogens may remain dormant, with no reproductive activity within soil or aquatic environments, while other pathogens can multiply in the environment. For example, non-reproducing *Leptospira* spirochetes can persist for several months in soils in the absence of a mammalian host [25]. A causative agent of cattle disease, *M. bovis* persists for long periods and may replicate in sandy soils used as bedding under certain conditions [22]. CWD-causing prions can also survive for long periods in soil [26]. In each case, the role of environmental factors in governing pathogen persistence, such as soil alkalinity, moisture, or specific mineral content, are poorly understood. 

Empirical data are needed to assess the time constants of processes involved in indirect pathogen transmission (e.g., replication rates in the environment; half-life decay rates of LIZs), as well as to parameterize transmission models. In the case of soil-borne *B. anthracis*, recent studies on the environmental conditions that support pathogen persistence include ecological niche modeling [37,38,39]), host seasonal resource selection in risk areas [40], host foraging behavior at LIZs [11,41], seasonal changes in host diet [12], and seasonal fluctuations in host antibody titers to *B. anthracis* [42]. These studies allow us to parameterize tactical models that estimate the force of infection (within or between species) within an anthrax transmission season on a single landscape. The long-lived nature of LIZs, however, demands the formulation of strategic models able to reliably estimate the number of “offspring” LIZs generated by a “parent” LIZ and to quantify the force of infection (i.e., persistence) across multiple seasons or years. Similarly, in the case of the water-borne bacterium *Vibrio cholerae,* the causative agent of cholera, sufficient data exist [26,37,40] to construct strategic models that account for transmission enhancement and seasonal affects through *V. cholerae* associations with zooplankton [23,24,41,42]. Likewise, a growing number of studies find that some bacterial pathogens, including *B. anthracis* [43], *B. thuringiensis* [44], *Salmonella enterica* [45], and *Escherichia coli* [46], may replicate in the rhizosphere or directly on plants. In addition to providing resources for replication, such environmental reservoirs may promote alternative pathogen transmission routes.

## 2. Host Interaction with Local Infectious Zones (LIZs) at the Landscape Level

In the 19th century, Louis Pasteur identified carcass sites as key to anthrax transmission [47], while 45 years ago Van Ness [48] proposed that under certain conditions, *B. anthracis* maintains high population densities in areas where it multiplies in the environment. Observations of naturally occurring carcass sites in Etosha indicate that spores persist for at least several years after carcass decomposition [11,49]. Environmental reservoirs may be influenced by nutrient availability, weather patterns, and bio-geo-chemical parameters [43,48,50]. Features of the exosporium can affect the ability of *B. anthracis* spores to bind to different soil types [51,52]. Thus, the rate at which a spore pool decays likely depends on local conditions [9]. CWD prion persistence and infectiousness also varies with soil type [26] and consequences of this variation in environmental persistence was examined with disease modeling [10]. In addition to physical and chemical variables affecting spore persistence, biological interactions between carcass materials and other species occurring in environmental reservoirs may alter the exposure of animal hosts to pathogens [53]. For example, hosts may be attracted to the growth and quality of vegetation that arises from nutrient deposition from carcasses [54,55,56,57]. Using camera traps, host visitation and duration rates at LIZs were quantified in the field for anthrax [11], *M. bovis* [58] and CWD [59]. These data are readily incorporated into disease models. Landscapes that support LIZ persistence can be characterized using habitat mapping procedures [37,40]: in the case of *B. anthracis* at continental [37,39,60,61] and local [40,62] scales. Similar approaches were employed for cholera [63] and brucellosis [64]. Kracalik et al. [65,66] used logistic regression approaches to estimate anthrax risk and Osnas et al. [67] employed a hierarchical Bayesian approach to produce spatially explicit estimates of prevalence for a wildlife CWD outbreak.

### 2.1. Local Infectious Zone (LIZ) Ecology

In the case of anthrax, seasonal peaks in disease incidence are commonly observed [9,12,51]. For example, major anthrax epizootics follow rain events or seasonal changes in green-up trajectories [19], particularly in grass [68] and shrubland [69]. Due to increased nutrient availability, plant growth at LIZs may outpace growth in non-LIZ areas, potentially promoting host foraging. In a field experiment, *B. anthracis* spores substantially increased the rate of establishment for a native grass, which was also taller when treated with blood [70]. Tall lush growth of plants resulting from a combination of the influence of the bacterium and input of nutrients from decaying carcasses may be attractive to grazing hosts and promote disease transmission [70]. Additionally, host populations may track vegetation responses to environmental triggers and migrate locally, concentrating susceptible hosts in areas where they experience high contact rates with LIZs. Likewise, individuals may change diet preferences seasonally to plants that are greening up. Prevalence rates of *Brucella* or *M. bovis* also tend to peak seasonally, with transmission occurring where host populations commingle [71,72]. Unlike *B. anthracis*, these two pathogens exhibit relatively short-term survival in the environment and thus are likely to have only intra-seasonal LIZ persistence. 

### 2.2. Host Movement Ecology and Transmission

For successful indirect transmission, hosts must contact LIZs. Estimating contact can be done across spatial scales and levels of resource selection as defined by Johnson [73]. As a first estimate of where contact may occur, one can estimate seasonal home ranges for susceptible hosts and compare those to environments that promote LIZ persistence, as was recently done for anthrax in elk (*Cervus canadensis*) [40] and bison (*Bison bison bison)* [74]. When modeling the likelihood of host presence in a LIZ region, we are most interested in local utilization distributions (UDs), where space is quantified in some form of density of animal positions over time [75,76]. Several techniques are available for quantifying these local UDs [75] in space and time [77]. New movement analysis tools, coupled with high resolution spatio-temporal data from GPS collars, allow us to directly estimate how individual re-visitation (spatial) and duration of visits [77] (spatio-temporal) to specific areas change seasonally. Such estimates can be used to parameterize movement patterns of hosts and related to LIZ concentrations. Most recently, tools were introduced to evaluate optimal parameter settings for the T-LoCoH (http://tlocoh.r-forge.r-project.org/) package for R, where home ranges and these visitation/fidelity metrics can be calculated, including examples of host/LIZ overlap in Etosha National Park (ENP), Namibia [78,79].

Seasonal and inter-annual variation in observed incidence are driven by factors affecting transmission itself, as well as factors affecting data-gathering. Anthrax incidence in Etosha, for example, is affected by LIZ demography and distribution, animal exposure to LIZs, and the immunological state of individuals [42,80]. Within the context of Johnson [73], third order selection (patch use within a home range), such as individual foraging or interactions at LIZs, can be measured using camera traps set at LIZs, as was done in ENP [11]. Observed anthrax incidence, however, differs from actual incidence because surveillance efforts are typically seasonal, though a modeling approach exists to account for this sampling bias [81]. These difficulties have been documented across disease systems, particularly in wildlife [34,82]. Thus, it is important to keep in mind how ecological and epidemiological processes interact with observation processes. For instance, anthrax outbreaks tend to be observed in Etosha after rainfall with a delay that could be explained by both ecological and observational mechanisms. Rainfall affects animal movement patterns, the splashing of spore-laden soil onto palatable grass leaves, and exposure to interacting microparasites and macroparasites [83,84]. However, there was also a significant correlation with the preponderance of wet season researcher activity [81]. In west Texas, anthrax in deer is also seasonal and has been correlated with seasonal peaks in vegetation greenup [19] and increased biting fly densities [34]. Also, case intensity may be amplified within an outbreak by blow flies [33]; the latter phenomenon can expand the zone of contamination at a LIZ during the first several days after host death.

### 2.3. Dynamic Thresholds and the Joint Modeling of Reservoir (LIZ) and Host Dynamics 

Individual host heterogeneities, along with geographical and environmental discontinuities, might critically amplify, dampen or lag the known effects of pathogen reservoirs. A recent study illustrated host population dynamics and environmental drivers are required to model anthrax outbreak periodicity [85], however the approach was not spatially explicit. Thus, continuity of geographical expansion and homogenous mixing of individuals are unsuitable assumptions for modeling many host-pathogen systems (see [86]). An approach that can account for such inhomogeneities, yet remains relatively simple, is to specify a low-dimensional model of the host, pathogen and reservoir dynamics that explicitly incorporates spatial and temporal lag effects. In reservoir-driven epidemics, considerable effort is currently being invested in unraveling host movement and behavior characteristics that shape the host’s susceptibility, thereby providing data for within-season models of outbreaks that can be coupled to an across-season model of the reservoir or LIZ-population dynamics. Such across-scale stochastic processes are often best characterized by a combination of a fine (fast and small) scale Brownian Motion (BM) process and a coarser scale pure-jump process [87,88], which combination can be often be adequately modeled as a Lévy process [89]. These models have been applied in various biological settings, including epidemiological data [86], with a primer provided here.

## 3. Discrete, Self-Decomposable (DSD) Parameter Estimation

Discrete, self-decomposable (DSD) stochastic processes [90,91,92] provide a way to combine short-term seasonal outbreaks of a number of pathogen systems (Table 1) modeled by a Lévy process with long-term gradual changes within LIZs modeled by a Brownian (i.e., Gaussian) processes. The first step is to define a stochastic process for the number of LIZs present within the smallest spatial and temporal resolution unit of the data (e.g., the 250 m^2^ pixel dimensions and 16-day NDVI measurements of the MODIS satellite system). The second step is to formulate a DSD probability generating function that is a composition of two distinct stochastic processes: a stochastic LIZ decay process and an “innovation” (random generation of new LIZs) jump process [91]. The resulting DSD process is then able to model jumps (various formulations can used [88,92]) in the LIZ sample path, as it accounts for abrupt changes in the number of LIZs within a pixel (noting that alternative, as well as accommodating LIZ persistence and LIZ “arrival” process parameters that are dependent on environmental covariates (thereby providing a natural test bed for the relevance of covariates). The arrival of naïve hosts to a pixel establishes a susceptible population, estimated from host movement data. At the population level, resource selection function-based probabilities can be used to define the likelihood a host will choose a pixel [40] (derived from GPS collar data and a use-available framework to model resource selection [93]), while visitation and duration metrics can be used to estimate the length of stay and number of return visits to a given pixel [77] (applying the T-LoCoH metrics to GPS collar data, e.g., [78]). Estimating foraging activity at LIZs within the pixel then effectively provides an informed measure of exposure rate. Camera trap data can be used to estimate foraging activity within a pixel, as was applied in ENP [11]. 

If LIZ dynamics are modeled as a first-order Markov process, then maximum likelihood estimation allows the calculation of the mean number of LIZs remaining in any pixel after *d* time steps. In its simplest formulation, the number of LIZs over time follows a Poisson process that implicitly assumes the persistence process is temporally homogeneous, thereby neglecting the effect of the covariates and links to host movement. To make the model spatially explicit, one can model the LIZ decay (epidemiological recovery) and LIZ innovation (epidemiological incidence) processes using environmental covariates in resource selection functions. To account for temporal heterogeneity in the abundance of LIZs, the well-known derivation of the Negative Binomial as a conditional Poisson distribution can be used, where the innovation rate itself is Gamma distributed [94]. The mean of this Gamma process can be made a function of environmental covariates. Then, LIZ persistence can be modeled using a combination of a deterministic trend (e.g., exponential decay) and random fluctuations due to environmental variation. Other elaborations are possible to make this approach more realistic, depending on the quantity and quality of data available to support identification and selection of models with additional complexity [95].

Estimating the probability of a threshold condition for disease emergence, a DSD model can be seeded with a single LIZ within a pixel in an otherwise “clean” landscape. Simple calculations using the model probabilistic structure can then be used to obtain explicit expressions of the average number of newly generated LIZs within any time frame: either after a single iteration of the process, or at the end of a season. Hence, the DSD model is a means to obtaining a “within-year” LIZ reproduction number (R_0_) as well as assessing the expected number of LIZs that remain on a landscape of a given size after one or more seasons (years). In this way, a DSD model connects the beginning of a given year’s zoonotic season with past dynamics, thereby providing a spatially explicit estimate of R_0_. With this quantitative framework in place, hypothetical “what-if” games simulating different control measures can lead to informed estimates of their effects. For example, carcass burning or burial (individual LIZ destruction) is a primary means of anthrax control during an epizootic. A DSD model can be used to simulate such a removal process by reducing the increase in LIZs between years and evaluating its impact on R_0_. A second type of control would be to evaluate the impact of vaccinating a host population, such as a bison herd in Montana [82], on the expected value of R_0_. These first two kinds of simulations would essentially assess changes to the rate of new LIZ formation due to healthy bison encountering fewer current LIZs (affected by carcass control) or reduced susceptibility (affected by vaccine coverage and efficacy). A third approach to control would be to evaluate the effect of excluding naïve hosts from pixels with LIZs. This would mimic the management strategy of using fences around pastures to keep livestock herds (such as bison in Montana) from areas of known historical outbreaks. Such exclusion would eliminate LIZ contacts by limiting the spatio-temporal jump processes to only non-excluded pixels, thereby lowering the average of the innovation process in the DSD model. The DSD framework provides a way to use empirical data to conduct plausible simulations relating to control strategies that are not possible to directly evaluate on real landscapes, but are likely to inform decisions, with applicability to several pathogens (Table 1).

## 4. Conclusions

The emergence of diseases caused by *environmentally-maintained indirectly transmitted pathogens* depends upon local and landscape-level variables, complicating disease modeling efforts. The analytical approaches synthesized here capitalize on advances in our knowledge of pathogen persistence, and high-resolution host movement and foraging behavior to estimate the basic reproductive number, R_0_ for such pathogens, using LIZs or patches of infection on the landscape. We illustrate that data are required from each the host population, the pathogen population, and the LIZs, as a separate and integral part of the modeling process. Data collection for such an approach should include monitoring and measurement of each population.

This quantitative framework is needed by real world stakeholders of agricultural and wildlife resources for them to manage environmentally transmitted diseases. As an example, anthrax is a globally occurring disease presenting a wide range of control challenges. Eradication of anthrax in Etosha for example, could negatively impact predator/scavenger populations [96], while eradication in commercial bison herds in Montana is highly desirable [82]. Modeling the effects of control in these systems also applies broadly to other landscapes, such as northern Canada, where anthrax threatens the survival of the endangered wood bison [97], and the Republic of Georgia, where livestock and human anthrax is re-emerging [68,98]. However, this modeling strategy is not anthrax specific, and can be extrapolated to other disease systems with environmentally-mediated indirect transmission, such as cholera, chronic wasting disease [27], brucellosis [71], or bovine tuberculosis [99], which are all associated with significant disease risk in multiple hosts, including humans. 

## Figures and Tables

**Table 1:** Several important diseases caused by pathogens (including bacteria, fungi, prions, and parasites) with environmentally maintained reservoirs.

Disease	Pathogen	Host	Environmental Reservoir	Local Infectious Zone (LIZ)	Landscape Characteristics	Survival Time in Environment	References
Anthrax	*Bacillus anthracis*	Wildlife and livestock	Host, bones, soil, water, vegetation	Carcass site, water’s edge	Grasslands, scrub/pothole regions	>1 year	[9]
Botulism	*Clostridium botulinum*	Birds & mammals	Host, honey, soil	Carcass site, honeybee colony	Cosmopolitan	>1 year	[21]
Bovine mastitis	*Mycoplasma bovis*	Bovids	Host, soil and/or animal bedding	Bedding within feedlot	Broad conditions	~1 year (needs futher study)	[22]
Brucellosis	*Brucella* spp.	Wildlife and livestock	Host, soil and/or birthing tissues, aborted fetuses	birthing tissues and aborted fetuses		~20–80 days (needs further study)	[16]
Cholera	*Vibrio cholerae*	Humans	Host, feces, zooplankton, saltwater	Estuaries	Periurban, coastal regions		[23,24]
Leptospirosis	*Leptospira* spp.	Animals, humans	Host, grass, moist soil, water	Grasslands, streams, rivers, ponds, lakes	Periurban, contaminated lakes		[25]
Chronic wasting disease	Prions	Cervids	Host, some soils	Salt/mineral sites, wallows	Host range & soils overlap		[26,27,28]
White-nosed syndrome	*Psuedogymnoascus destructans*	Hibernating bats	Host, some soils	Bat hibernacula	Cave system or mountain range		[29,30]
Toxoplasmosis	*Toxoplasma gondii*	Mammals	Host, feces, soil, invertebrates	Soils, streams, bays, estuaries	Periurban areas, coastal regions		[31,32]
